# Mechanism of ERK/CREB pathway in pain and analgesia

**DOI:** 10.3389/fnmol.2023.1156674

**Published:** 2023-03-16

**Authors:** Weizhe Zhen, Hongjun Zhen, Yuye Wang, Leian Chen, Xiaoqian Niu, Bin Zhang, Ziyuan Yang, Dantao Peng

**Affiliations:** ^1^Graduate School, Beijing University of Chinese Medicine, Beijing, China; ^2^Department of Neurology, China-Japan Friendship Hospital, Beijing, China; ^3^Department of Orthopaedics, Handan Chinese Medicine Hospital, Handan, Hebei Province, China; ^4^Graduate School, Chinese Academy of Medical Sciences and Peking Union Medical College, Beijing, China; ^5^Graduate School, Peking University China-Japan Friendship School of Clinical Medicine, Beijing, China

**Keywords:** ERK/CREB pathway, pain, analgesia, nervous system, adverse effects

## Abstract

Research has long centered on the pathophysiology of pain. The Transient Receiver Potential (TRP) protein family is well known for its function in the pathophysiology of pain, and extensive study has been done in this area. One of the significant mechanisms of pain etiology and analgesia that lacks a systematic synthesis and review is the ERK/CREB (Extracellular Signal-Regulated Kinase/CAMP Response Element Binding Protein) pathway. The ERK/CREB pathway-targeting analgesics may also cause a variety of adverse effects that call for specialized medical care. In this review, we systematically compiled the mechanism of the ERK/CREB pathway in the process of pain and analgesia, as well as the potential adverse effects on the nervous system brought on by the inhibition of the ERK/CREB pathway in analgesic drugs, and we suggested the corresponding solutions.

## 1. Introduction

Pain is a highly crucial bodily sensation that not only causes people unending suffering but also has a significant impact on many of their everyday activities. Our comprehension of pain is likewise becoming more thorough as research into its mechanism continues to advance (Fitzcharles et al., 2021). Numerous rigorous researches on the pathogenesis of pain have identified numerous receptors, pathways and approaches that are intimately connected to the pathogenesis of pain. The TRP protein family is the most well-known of them. Researchers have confirmed that the ERK/CERB pathway, sodium channel protein, calcium channel and dmPFC/vlPAG pathway are also strongly associated to the mechanism of pain in addition to the TRP protein family (Basbaum et al., 2009; Yin et al., 2020). The study of the relationship between the ERK/CREB pathway and the pathophysiology of pain has steadily emerged as a research hotspot. In-depth research on numerous analgesic medicines that target the ERK/CREB pathway is being done, although these treatments could have unfavorable side effects. The association between the ERK/CREB pathway and the mechanism of pain and analgesia, as well as the adverse effects of analgesic medications targeting the ERK/CREB pathway are currently lacking in systematic summaries and reviews. Therefore, we summarized the mechanism of ERK/CREB pathway in the pathogenesis of pain and analgesia from the three aspects of oxidative stress and neuroinflammation, neurodegeneration mediated by neuronal excitatory toxicity and activation of astrocytes and microglia, MicroRNA-mediated ERK/CREB pathway and discussed the adverse effects of analgesic therapies targeting ERK/CREB pathway related to nervous system mainly including the decline of cognitive learning ability, mental and psychological abnormalities and the obstruction of nerve regeneration and summarized methods to solve the problems.

## 2. ERK/CREB pathway

Extracellular regulated protein kinases (ERK), a member of the family of mitogen-activated protein kinases (MAPK), have the crucial role of transmitting surface receptor signals to the nucleus (Wang and Mao, 2019). The ERK family currently known members are ERK1, ERK2, ERK3, ERK4, and ERK5. As a transmitter of cell signals, ERK has a significant role in a variety of fundamental cell functions, including cell migration, differentiation, growth, and survival (Lavoie et al., 2020). In the past decades, studies on the relationship between ERK and diseases have shown that ERK is involved in the onset and progression of cancer, Alzheimer’s disease and AIDS (Fang and Richardson, 2005; Du et al., 2019; Yang et al., 2020). Recent studies have revealed that ERK pathway is significantly up-regulated in the Warburg effect, which is closely related to the metabolism of tumor cells. In parts of the brain that are intimately associated with depression, the ERK pathway is markedly downregulated (Wang and Mao, 2019; Cha et al., 2021). It has also clarified the important role of blocking ERK pathway in the treatment of pancreatic ductal adenocarcinoma (PDAC; Bryant et al., 2019) and also found that ERK has a two-way regulatory role in the pathophysiological of cardiac hypertrophy (Gallo et al., 2019).

CREB refers to cAMP-response element binding protein which can selectively bind cAMP-response element (CRE) and has the function of regulating the transcription of multiple cell genes including dopaminergic neurons. Recent studies have shown that CREB activation can play a harmful role in mediating drug addiction, anxiety-like behavior, insulin resistance, leukemia and other processes or diseases (Carlezon et al., 2005; Tregnago et al., 2016; Yoon et al., 2021) while CREB activation under different conditions can also improve muscle performance. It was shown that Sirt6 downregulates Sox6, a key blocker of slow fiber-specific genes, by increasing CREB transcription, which in turn improves muscle performance (Song et al., 2022). CREB activation can also combat obesity related metabolic diseases, promote the expression of memory-related genes, play a neuroprotective role and even play an antidepressant role (Walton and Dragunow, 2000; Blendy, 2006; Kim et al., 2019). The decreased expression of CREB may also be related to the pathophysiological process of heart failure. It was shown that disruption of Gsα reduced CREB1 expression and inhibited Bmp10-mediated signaling pathways, which in turn deteriorated cardiac function and induced severe cardiac remodeling (Yin P. et al., 2021). The process of ERK/CREB pathway and its role in some important physiological processes are gradually being discovered and highly concerned by researchers. Protein kinase A (PKA) induced serine-133 phosphorylation of cAMP response element binding protein (CREB) through extracellular signal-regulated kinase (ERK; Otani et al., 2018). Feng et al. found the close relationship between the inhibition of ERK/CREB pathway and the pathogenesis of post-traumatic stress disorder (PTSD) through experiments, and also revealed the close relationship between the activation of ERK/CREB pathway and the improvement of neuronal plasticity (Feng et al., 2020). PTSD is one of many psychosocial diseases closely related to the inhibition of ERK/CREB pathway. Other psychosocial diseases related to ERK/CREB pathway include depression, etc. (Moriguchi et al., 2020). In addition, this pathway is also associated with neurodevelopmental processes and some neurodevelopmental disorders (Servili et al., 2020).

## 3. The mechanism of ERK/CREB pathway in pain and analgesia

The ERK/cREB pathway is another important biochemical mechanism involved in the process of pain in addition to the TRP family. Numerous studies have investigated in-depth the connection between the ERK/cREB pathway and pain and analgesia (Cao et al., 2012).

### 3.1. Oxidative stress and neuroinflammation

BDNF-TrkB-ERK-CREB pathway is widely involved in many processes related to the nervous system. BDNF-TrkB-ERK-CREB pathway is closely related to oxidative stress and neuroinflammatory process (Zhang et al., 2020). Li et al. found that the increase of pain and pain aversion in rats with bone cancer pain would be accompanied by the up-regulation of BDNF expression. The phosphorylation of ERK/CREB pathway (pERK/pCREB) will be up-regulated with the aggravation of pain and pain aversion or exogenous injection of BDNF into rats suffering from bone cancer pain. After blocking BDNF-rkB signal, the expression level of pERK/pCREB decreased (Li et al., 2022). This demonstrates that the ERK/CREB pathway is crucial for the BDNF-TrkB signal-mediated pain and pain aversion. Furthermore, it suggests that ERK/CREB pathway may induce pain by mediating oxidative stress and neuroinflammatory processes (Espinosa et al., 2006).

Consistent with this, the silencing of PAR-2 can reduce the pain and inhibit the activation of astrocytes and activated ERK/CREB pathway in rats with cancerous bone pain, revealing that the silencing of PAR-2 inhibits the activation of ERK/CREB pathway and astrocytes, thus reduces the pain of rats (Tang et al., 2022). This further demonstrates that blocking the ERK/CREB pathway can have analgesic effects.

### 3.2. Neurodegeneration mediated by neuronal excitatory toxicity and activation of astrocytes and microglia

The phosphorylation of ERK and CREB was closely related to NMDARs (Cao et al., 2012). Researchers found caveolin-1 can directly combine with NR2B to activate the ERK/CREB signal pathway in a NMDA receptor 2B subunit (NR2B) dependent manner to mediate central sensitization and induce pain, while increasing the intracellular Ca^2+^ concentration in the anterior cingulate cortex (Yang et al., 2015). Further research demonstrated that the NMDAR-CaMKII-ERK/CREB signal pathway can activate microglia and astrocytes, which can cause excitatory toxicity in neurons, cause neurodegeneration, and result in neuropathic pain (Tsuda and Inoue, 2016; Iyaswamy et al., 2018). This also suggests that neuronal excitatory toxicity and neurodegeneration mediated by activation of astrocytes and microglia are important molecular mechanisms and pathological processes of pain.

In terms of analgesia, inhibition of ERK/CREB pathway can exert analgesic effect by reducing neuronal excitability. In the rat model of neuropathic pain induced by spinal nerve ligation, the antagonist of calcium channel SOCCs YM-58483 can reduce the level of P-ERK and P-CREB, and also inhibit the release of IL-1β, TNF- α, PGE2 in the spinal cord, and then exert the analgesic effect, reveals that its analgesic mechanism may be to inhibit the ERK/CREB pathway of neurons, reduce the release of central IL-1β, TNF- α, and PGE2, thereby reducing the excitability of neurons in the spinal dorsal horn (Qi et al., 2016).

### 3.3. MicroRNA-mediated ERK/CREB pathway

In the process of pain, the activation of ERK/CREB pathway is often mediated by MicroRNA. In rats with chronic inflammatory visceral pain, MiR-211 can target the 3’-UTR of the ERK gene. This greatly raises the expression level of ERK and CREB, promotes visceral hypersensitivity, and then produces pain (Sun et al., 2019).

Consistently, MicroRNA-365 can reduce β- Expression of arrestin2, ERK, CREB proteins, negative targeting β- Arrestin2, which inhibits the activation of ERK/CREB pathway, and then reduces the tolerance to morphine analgesia, and plays a better analgesic effect (Wu et al., 2018).

## 4. Analgesics designed based on inhibition of ERK/CREB pathway

Some clinical analgesics and pharmacological studies of new analgesics have targeted the ERK/CREB pathway and performed an analgesic role by inhibiting the ERK/CREB pathway due to the crucial role of the ERK/CREB pathway in the molecular mechanism of pain control (Choi et al., 2009). Both the analgesic drugs targeting ERK/CREB pathway that have been put into clinical use and the new drugs targeting ERK/CREB pathway that are being studied will play an important role in clinical pain control. Lidocaine effectively treats neuropathic pain by drastically lowering the ERK 1/2 protein and CREB protein of neuropathic rats to 39 and 48%, respectively, and the mRNA level of pro-inflammatory cytokines (Joo et al., 2011). It has also been discovered that the active ingredients of some natural plants or Chinese medicines can function as an analgesic by blocking the ERK/CREB pathway. Intraperitoneal injection of oxymatrine can significantly lower the expression of p-ERK and p-CREB protein, inhibit the activation of the ERK/CREB pathway, and have analgesic effects in a model of chronic neuropathic pain caused by chronic constrictive injury of the sciatic nerve (CCI; Wang et al., 2013). As one of the main extracts of Chinese medicine fructus cnidii, osthol quickly suppressed the ERK/CREB pathway in neuropathic pain model mice, as well as the P2Y1 receptor-dependent JNK signaling pathway and then alleviate the mechanical abnormal pain (Li et al., 2020).

In addition to neuropathic pain, migraine symptoms can be treated with analgesics that target the ERK/CREB pathway. BAY-117082, an NLRP3 inflammatory body inhibitor, can significantly reduce the expression of p-ERK, p-CREB, p-Akt, and p-PI3K to inhibit the ERK/CREB pathway and greatly lower the expression level of NLRP3 complex components such as IL-1β, IL-18 that play a role in alleviating pain attack and hyperalgesia in migraine mice (Filippone et al., 2022).

## 5. Adverse effects of analgesics related to nervous system

Analgesic methods targeting ERK/CREB pathway are not only one of the focuses of basic research, but also more and more used in clinic. However, increasing numbers of research have demonstrated that analgesic strategies that target the ERK/CREB pathway may have adverse effects on a variety of systems, including the nervous system, as a result of ERK/CREB suppression (Guo et al., 2017; Gong et al., 2022). We outlined the adverse effects on the nervous system of analgesic techniques that target the ERK/CREB pathway, as well as their mechanisms.

### 5.1. Adverse effects of analgesics on ERK/CREB pathway on cognitive and memory functions

The ERK/CREB pathway can now be inhibited by a number of analgesics used in clinical practice and novel analgesics under investigation. More and more research findings imply that the cognitive and memory skills may be impacted by suppression of the ERK/CREB pathway. In the experiment, Fatahi et al. discovered that rats given morphine had a markedly decreased ability to activate CREB (Fatahi et al., 2020). What is more important, repeated morphine administration could block the activation of CREB and impair the cognitive function of mice (Guo et al., 2019). In order to further confirm that the inhibition of ERK/CREB pathway will lead to the decline of cognitive and memory functions, researchers have carried out more in-depth mechanism research. Through Western blot, Coinmunoprecipitation, Biotin switch assy, Immunofluorescence, and other experiments, Zhang et al. discovered that the interaction of nNOS-CAPON may result in the inhibition of the downstream ERK-CREB pathway in the Alzheimer’s disease mouse model, causing excitotoxicity and abnormal dendritic spine development, affecting cognitive function and triggering the progress of Alzheimer’s disease (Zhang Y. et al., 2018). PS1 deficiency in presenilin 1 (PS1) knocked out human neural stem cells (NSCs) based on CRISPR/Cas9 system can also lead to autophagy damage of human neural stem cells by down-regulating ERK/CREB signal pathway, thus exacerbating the process of Alzheimer’s disease (Chong et al., 2018). In cortical neurons treated with Aβ1-42 and APP/PS1 mice, striatum-rich phosphatase 61 (STEP61) also damaged the formation of long-term memory by inhibiting ERK/CREB pathway (Zhang et al., 2013).

Additionally, some studies have shown how crucial the ERK/CREB pathway is in triggering long-term potentiation and fostering the formation of memories (Nabavi et al., 2014). Through the use of western blot, electrophysiological recording, immunohistochemistry, and other techniques, numerous researchers have discovered that the activation of the ERK/CREB pathway is crucial for inducing long-term potentiation (Xin et al., 2006; Cao et al., 2009). Further study is required to determine whether inhibiting the ERK/CREB pathway may result in anomalies that cause long-term potentiation and impact cognitive performance.

**Figure 1:**
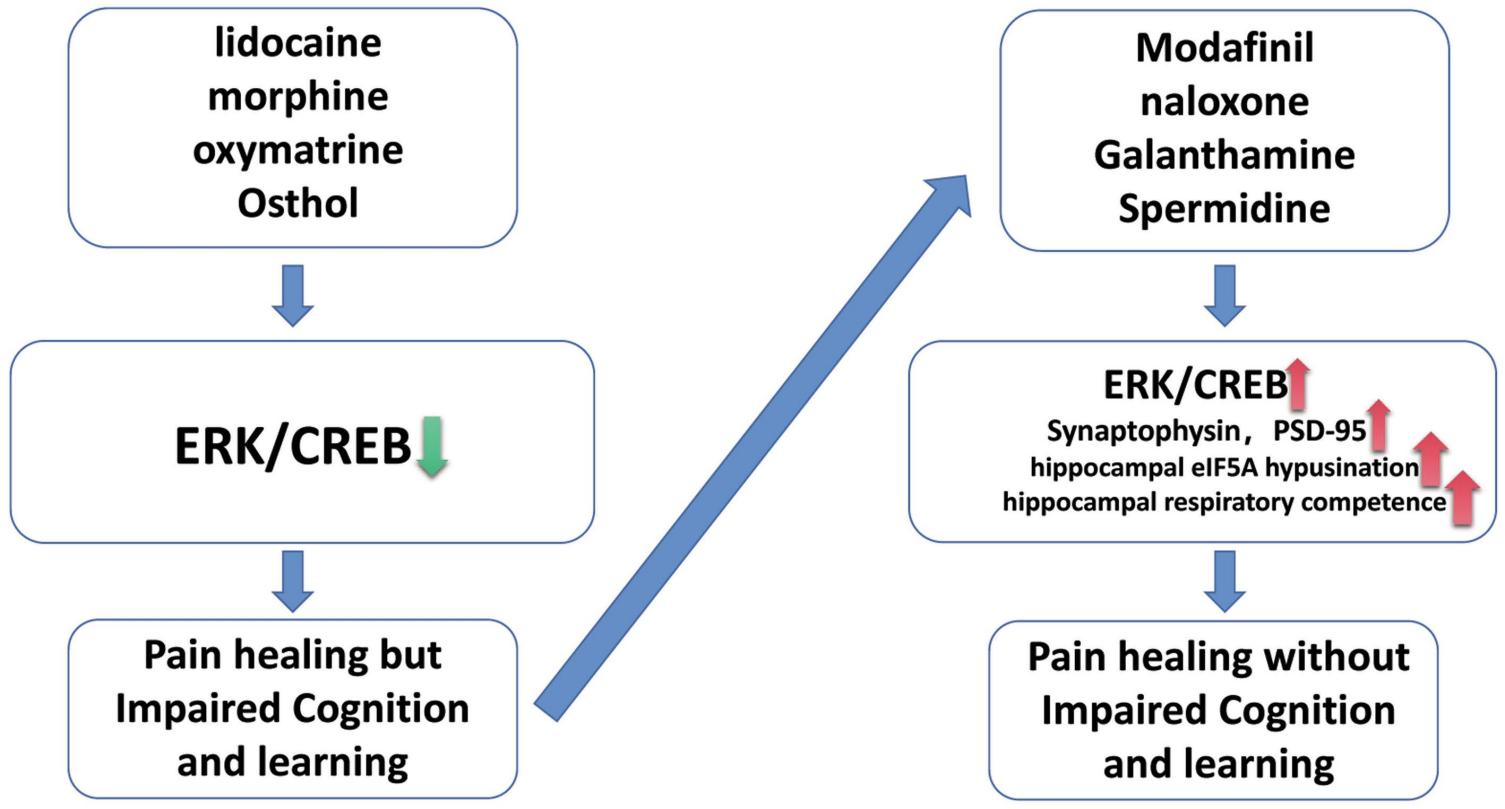
Some strategies for treating pain while avoiding cognitive impairment.

Numerous researchers have developed various experimental programs and engaged in extensive study to address the impact of various analgesics on cognitive and memory skills, and they have finally produced some useful preventative strategies (Figure 1). First, some scientists discovered that activating the ERK/CREB pathway can prevent analgesic medicines from damaging cognition and memory. The patient’s compromised cognitive and memory functions may be saved if the ERK/CREB pathway is moderately activated to protect it from over-inhibition by analgesic medicines as the patient’s pain continues to subside or disappears. Tropomyosin associated kinase B (TrkB) can be phosphorylated to activate the ERK/CREB pathway and enhance the early phenotypic of mice with an AD-like phenotype. Additionally, the TrkB receptor’s signaling pathway is crucial for neuronal development and synaptic plasticity (Fan et al., 2020). By turning on the ERK/CREB pathway in the medial prefrontal cortex, low-dose modafinil can likewise prevent the behavioral and synaptic harm brought on by persistent morphine use (Yin et al., 2022). Fatahi et al. also found that naloxone, a morphine receptor antagonist, can play a neuroprotective role by activating CREB and save cognitive brain function damage (Fatahi et al., 2020). Secondly, we can try to save the impairment of cognitive and learning function caused by analgesics through other pathways and approches. Galantamine and donepezil are both cholinesterase inhibitors and are used for the treatment of mild cognitive impairment. Recent studies have shown that galanthamine can improve the cognitive function of model mice by reducing the inflammatory response of hippocampal neuron system in Alzheimer’s disease mice and alleviating the loss of hippocampal synapse-related proteins (SYN and PSD-95) induced by lipopolysaccharide (Liu et al., 2018). However, whether the simultaneous use of analgesics and galanthamine can simultaneously exert analgesic effects and improve cognitive function, and ensure high safety, still needs further research. In addition to the combination of drugs, we can also try to alleviate cognitive learning function by adjusting diet. Tryptophan is an essential amino acid that requires a person to consume it from the diet. Studies have shown that a diet rich in tryptophan can compensate for age related decline in social- cognitive processes (Zamoscik et al., 2021). Through experiments, researchers found that spermidine can pass through the blood–brain barrier of mice, boost the utilization rate of eIF5A and mitochondrial function in the hippocampus, and also enhance the spatial learning ability. In the aging Drosophila model, spermidine can enhance mitochondrial respiratory capacity and promote the improvement of learning function with the participation of autophagy regulator Atg7 and mitotic mediators Parkin and Pink1 (Schroeder et al., 2021). Although such a strategy has relatively higher safety, but it may take a period of time to achieve certain results (Du et al., 2017).

### 5.2. Adverse effects of analgesics based on ERK/CREB pathway on psychosis

Psychosis is intimately correlated with the ERK/CREB pathway, particularly depression (Liu et al., 2022). In the study of the molecular mechanism related to prenatal stress and the occurrence of depression in the offspring, the activation of ERK/CREB was inhibited in the offspring depression mice, revealing the close relationship between ERK/CREB pathway and the occurrence of depression (Guan et al., 2013). Further research shows that, glycogen synthase kinase-3 (GSK-3) can hasten the progression of depression by blocking the ERK/CREB pathway (Peng et al., 2018). This explicitly explains how blocking the ERK/CREB pathway contributes to the onset and progression of depression. Depressive disorders frequently coexist with neurological diseases. Its mechanism has also been proved to be related to ERK/CREB pathway. In cerebral ischemia rats model, the levels of p-ERK and p-CREB were significantly decreased, the activation of ERK/CREB pathway was significantly inhibited, and the expression of pro-inflammatory cytokine IL-6 and TNF-α was increased in post-stroke depression through sucrose preference test (SPT), forced swimming test (FST), tail suspension test (TST), western blot and Elisa experiments (Yin Q. et al., 2021). It was also found in epileptic rats that the development of depression comorbidity was related to the decrease of ERK2 expression and the inhibition of CREB phosphorylation (Sadeghi et al., 2021). However, endogenous opioid drugs lead to depression through multiple mechanisms including preventing the ERK/CREB pathway from being activated. Some researchers systematically analyzed the anxiety caused by the increase of dynorphin level and proposed the role of ERK/CREB pathway in it (Bali et al., 2015).

Researchers are currently exploring drugs that can reduce depressive symptoms by activating ERK/CREB. Through the forced swimming test, autonomous activity test, and western blot, the antidepressant effect of inosine was clarified. Inosine can contribute to the reduction of depressive behavior by activating the ERK/CREB pathway in the hippocampus and prefrontal cortex (Yuan et al., 2018). For patients with depression due to the use of analgesics, whether or not the analgesics they use act on ERK/CREB pathway, they can use some drugs appropriately after the pain symptoms are relieved to activate the ERK/CREB pathway and relieve the depressive symptoms. In addition to activating the ERK/CREB pathway, we also have many commonly used antidepressants to choose from, such as fluoxetine, sertraline, etc. At present, there are no drug test results against the combination of these antidepressants and analgesics. However, it would be ideal if more thorough clinical trials could back up its clinical efficacy and safety when used with analgesics. In addition to antidepressant drugs in western medicine, there are also some traditional Chinese medicine treatments that can be used to alleviate the mental and emotional problems caused by analgesics, such as Xiao-Yao-San Frmula, Jiao-Tai-Wan Formula and acupuncture. Its mechanism has also been gradually clarified by researchers (Jung et al., 2021; Liu X. et al., 2021).

### 5.3. Adverse effects of analgesics based on ERK/CREB pathway on nerve regeneration

Activation of ERK/CREB pathway is an important molecular mechanism for promoting nerve regeneration after nerve injury (Jiao et al., 2005). it was discovered that downregulation of motoneuron-specific molecules was linked to the activation of the ERK/CREB pathway, which was crucial for the regeneration of motor neurons in rats with facial axonotomies (Ishijima and Nakajima, 2022). Seo et al. found that, ERK/CREB pathway was inhibited in the model rats with sciatic nerve injury, while exercise training and bone marrow stromal cell implantation could significantly activate ERK/CREB pathway and then promote nerve regeneration (Seo et al., 2021). This process most likely shares a tight relationship with Schwann cell signal transduction (Ulrichsen et al., 2022). The ERK/CREB pathway must also be activated for the nerve regeneration process to take place following a cerebrovascular disease-related nerve injury. In the ischemic stroke mouse model, it was discovered that overexpressing Ras-related C3 botulinum toxin subtype 1 (Rac1) increased peripheral cell protein levels and blood vessel density around the infarction, and that inhibiting Rac1 prevented the production of ERK1/2 and CREB, which then prevented nerve regeneration and nerve function recovery (Bu et al., 2019).

Activating the ERK/CREB pathway is also necessary for nerve regeneration following injury to the neuromuscular junction’s nerve endings. Damaged neurons can swiftly release ATP, cause ERK and CREB phosphorylation, activate the ERK/CREB pathway, promote nerve regeneration, and restore neuronal function (Negro et al., 2016). In the process of nerve regeneration, the activation of ERK/CREB pathway also requires the participation of TRP protein family, which is also closely related to pain. The deletion of the TRPc1 gene greatly hindered the activation of the ERK/CREB pathway and prevented neuron regeneration (Du et al., 2017). This also directly revealed that the inhibition of ERK/CREB pathway would have adverse effects on the process of nerve regeneration.

To avoid the adverse effects on the process of nerve regeneration, some drugs or non-drug therapies can be used to save the over-inhibition of ERK/CREB pathway by analgesics. The heparin can promote nerve regeneration by up-regulating the expression of ERK/CREB pathway in the brain of rats during the rehabilitation process after cerebral ischemia and reperfusion injury, and then mediate the multiple effect protein (PTN)/ syndecan-3 pathway (Liu W. et al., 2021). The Chinese Medicine ingredient panaxydol can induce axonal growth and play a role in nerve regeneration by activating ERK/CREB pathway (Li et al., 2018). Some non-pharmaceutical therapies are increasingly valued by researchers because of their advantages such as low incidence of adverse effects. Through RNA interference, immunocytochemical analysis, protein blotting, and qPCR analysis, it was found that low level laser irradiation can activate the ERK/CREB pathway and enhance nerve regeneration, such as neuron differentiation, by raising the amount of calcium ion (Yan et al., 2017). The research focused on the molecular mechanism of photobiomodulation in promoting nerve regeneration and function enhancement was deeply studied for the non-invasive advantages of this treatment. Photobiomodulation can promote nerve regeneration by activating ERK/CREB pathway and then increasing the expression of BDNF (Heo et al., 2019). These drug therapy or non-drug therapy can be used when the pain feeling is relieved and the nerve regeneration disorder occurs at the same time, in order to save the excessive inhibition of ERK/CREB pathway. In addition, there are some active ingredients of Chinese medicine that can enhance nerve regeneration through different ways (Table 1), but whether they can be taken in conjunction with analgesics to avoid the inhibition of nerve regeneration remains to be further verified.

**Table 1:** Herbal drugs that promote nerve regeneration through other ways.

Drug	Disease	Model	Pathway/Target	Effect	Year	References
Rhodioloside	Spinal Cord Injury	Rats	HIF-1 pathway	Repair damaged neurons within spinal cord tissue	2020	Ha et al. (2020)
Total Glycosides of Cistanche deserticola	Ischemic Stroke	Rats	Nrf-2/Keap-1	Promote nerve regeneration	2020	Wang et al. (2020)
Panax notoginseng saponins	Ischemic Stroke	Rats	ROCKII	Promote synaptic regeneration	2021	Zhou et al. (2021)
Panaxydol	Sciatic Nerve Injury	Rats	BDNF	Increase the myelination of regenerated nerve fibers	2022	Wang et al. (2022a)
Acetyl-11-keto-beta-boswellic acid	Sciatic Nerve Injury	Rats	Phagosome pathway	Promote myelin sheath and axon regeneration	2022	Wang et al. (2022b)
Total astragalosides	Multiple Sclerosis	Mice	Wnt/β-catenin signaling pathway	Promote oligodendrocyte precursor cell differentiation and enhance remyelination	2022	Yuan et al. (2022)
Radix Astragalus Polysaccharide	Peripheral nerve injury	Rats	AKT/eNOS signaling pathway	Promote axonal regeneration and remyelination	2022	Zhang et al. (2022)

## 6. Discussion

The TRP protein family has historically received the majority of attention in our research on the mechanism of pain, while the ERK/CREB pathway and other closely related pathways have received far less attention. Systematic summaries and reviews of these studies are also lacking. At the same time, future research should pay more attention to the relationship between the activation of ERK/CREB pathway and TRP family in the process of pain. The activation of ERK/CREB pathway is closely related to the occurrence of pain, but the current research has not done more in-depth research on the downstream reaction and changes after the activation of ERK/CREB pathway, which is an important direction of our future research. Years of studies have revealed that aberrant mitochondrial structure and function are directly related to the pathogenesis of pain (He et al., 2020). Recent studies have shown that pain may be closely related to Parkin-mediated mitophagy, and researchers have also found that ERK/CREB pathway may also be related to mitochondrial membrane permeability changes, although more extensive investigations are needed to confirm these findings (Huang et al., 2014; Zhang Z. et al., 2018). Future research on the relationship between ERK/CREB pathway and the pathogenesis of pain should concentrate on the structural and functional damages of mitochondria. The pathogenesis of pain is also closely related to the immune processes of the body. The results of several experimental studies and bioinformatics analyses suggest that abnormalities in dendritic cells play a very important role in the pathogenesis of pain (Huang et al., 2016; Ye et al., 2022). In contrast, prostaglandin analogues induce the ERK/CREB pathway, which in turn leads to dendritic cell maturation (Pandey et al., 2017). Whether excessive activation of the ERK/CREB pathway leads to abnormalities in dendritic cells that cause pain deserves further experimental investigation by researchers.

**Figure 2:**
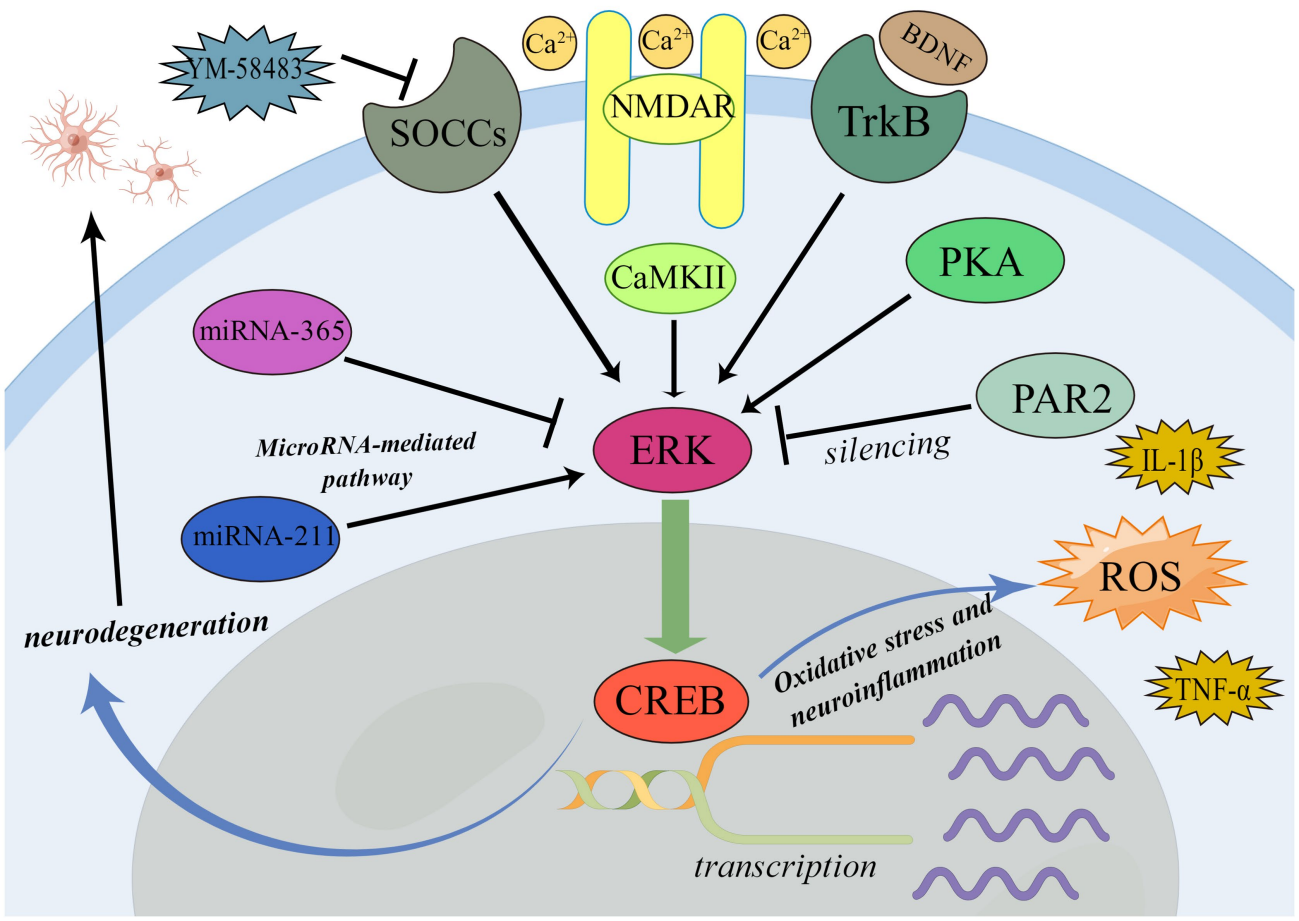
Mechanism of ERK/CREB pathway in pain and analgesia.

Since the ERK/CREB pathway can be inhibited and have analgesic effects, this pathway is the target of numerous current drugs and novel drugs being developed. However, the inhibition of the ERK/CREB pathway, according to a large number of basic experiments and clinical studies, will likely have adverse effects on the nervous system mainly including the decline of cognitive learning ability, mental and psychological abnormalities and the obstruction of nerve regeneration. We summarized in detail the mechanism of ERK/CREB pathway in pain and analgesia and the mechanism of adverse reactions of the nervous system related to analgesics based on ERK/CREB pathway (Figure 2; Table 2), and also proposed methods to solve and avoid adverse reactions of the nervous system. In order to fully come into clinical decision-making in the future and provide patients with more precisely targeted pain treatment, these methods, which are based on numerous experimental research, adequate analysis, and summary, nevertheless require further clinical trials for validation.

**Table 2:** The mechanism of adverse effects of the nervous system related to ERK/CREB pathway inhibition.

Model	Animal/cell	ERK/CREB pathway	Adverse effect	Mechanism	Year	Reference
nNOS-deficient mice and APP/PS1 mice	Mice	Inhibition	Cognitive and memory functions	Excitotoxicity and abnormal dendritic spine development	2019	Zhang Y. et al. (2018)
presenilin 1 (PS1) knocked out human neural stem cells (NSCs)	Human neural stem cells	Inhibition	Cognitive and memory functions	Autophagy damage of human neural stem cells	2018	Chong et al. (2018)
APP/PS1 mice and cortical neurons treated with amyloid β (Aβ)1–42 peptides	Mice and cortical neurons	Inhibition	Cognitive and memory functions	Related to α7 nicotinic acetylcholine receptors	2013	Zhang et al. (2013)
chronic stress mouse model	Mice	Inhibition	Psychosis	Related to BDNF	2018	Peng et al. (2018)
middle cerebral artery occlusion	Rats	Inhibition	Psychosis	Downstream of BDNF signaling	2021	Yin Q. et al. (2021)
epilepsy-associated depression	Rats	Inhibition	Psychosis	Related to BDNF and cFOS	2021	Sadeghi et al. (2021)
Axotomy of the rat facial nerve	Rats	Inhibition	Nerve regeneration	Downregulation of motoneuron-specific molecules	2022	Ishijima and Nakajima (2022)
sciatic nerve injury	Rats	Inhibition	Nerve regeneration	Schwann Cell Signaling	2021	Seo et al. (2021)
middle cerebral artery occlusion	Mice	Inhibition	Nerve Regeneration	Increased vascular density and the protein level of pericytes in the peri-infarct zone	2019	Bu et al. (2019)
TRPC1 knockout	Mice	Inhibition	Nerve regeneration	TRPC1 knockout abolished the EE-induced spatial memory enhancement, LTP induction, and neurogenesis in hippocampal DG subset	2017	Du et al. (2017)

## Author contributions

WZ, HZ, YW, LC, and DP collated, summarized and discussed the content of the review, and wrote the manuscript, WZ, XN, BZ, and ZY prepared figures and tables. DP supervised and revised the manuscript. All authors contributed to the article and approved the submitted version.

## Funding

This work was supported by National Key R&D Program of China (Grant No. 2022YFC2010103) and Central health research project (Grant No. 2020ZD10).

## Conflict of interest

The authors declare that the research was conducted in the absence of any commercial or financial relationships that could be construed as a potential conflict of interest.

The handling editor XL declared a shared affiliation with the authors WZ and DP at the time of review.

## Publisher’s note

All claims expressed in this article are solely those of the authors and do not necessarily represent those of their affiliated organizations, or those of the publisher, the editors and the reviewers. Any product that may be evaluated in this article, or claim that may be made by its manufacturer, is not guaranteed or endorsed by the publisher.
